# The association between transfer coefficient of the lung and the risk of exacerbation in asthma-COPD overlap: an observational cohort study

**DOI:** 10.1186/s12890-021-01815-w

**Published:** 2022-01-12

**Authors:** Hiroaki Ogata, Katsuyuki Katahira, Aimi Enokizu-Ogawa, Yujiro Jingushi, Akiko Ishimatsu, Kazuhito Taguchi, Hiroko Nogami, Hiroshi Aso, Atsushi Moriwaki, Makoto Yoshida

**Affiliations:** Department of Respiratory Medicine, National Hospital Organization Fukuoka National Hospital, 4-39-1 Yakatabaru, Minami-ku, Fukuoka, 811-1394 Japan

**Keywords:** Asthma-COPD overlap, Diffusing capacity of the lung, Transfer coefficient of the lung, Exacerbation, Forced expiratory volume in 1 s

## Abstract

**Background:**

Asthma–chronic obstructive pulmonary disease (COPD) overlap (ACO) patients experience exacerbations more frequently than those with asthma or COPD alone. Since low diffusing capacity of the lung for carbon monoxide (D_LCO_) is known as a strong risk factor for severe exacerbation in COPD, D_LCO_ or a transfer coefficient of the lung for carbon monoxide (K_CO_) is speculated to also be associated with the risk of exacerbations in ACO.

**Methods:**

This study was conducted as an observational cohort survey at the National Hospital Organization Fukuoka National Hospital. D_LCO_ and K_CO_ were measured in 94 patients aged ≥ 40 years with a confirmed diagnosis of ACO. Multivariable-adjusted hazard ratios (HRs) for the exacerbation-free rate over one year were estimated and compared across the levels of D_LCO_ and K_CO_.

**Results:**

Within one year, 33.3% of the cohort experienced exacerbations. After adjustment for potential confounders, low K_CO_ (< 80% per predicted) was positively associated with the incidence of exacerbation (multivariable-adjusted HR = 3.71 (95% confidence interval 1.32–10.4)). The association between low D_LCO_ (< 80% per predicted) and exacerbations showed similar trends, although it failed to reach statistical significance (multivariable-adjusted HR = 1.31 (95% confidence interval 0.55–3.11)).

**Conclusions:**

Low K_CO_ was a significant risk factor for exacerbations among patients with ACO. Clinicians should be aware that ACO patients with impaired K_CO_ are at increased risk of exacerbations and that careful management in such a population is mandatory.

**Supplementary Information:**

The online version contains supplementary material available at 10.1186/s12890-021-01815-w.

## Background

Over the last decade, the clinical characteristics of patients with coexisting asthma and chronic obstructive pulmonary disease (COPD), namely asthma–COPD overlap (ACO), have been matters of great concern for physicians [1, 2]. A meta-analysis of epidemiological studies revealed that more than a quarter of COPD subjects were compatible with ACO [3]. Since the diffusing capacity and transfer coefficient of the lung for carbon monoxide (D_LCO_ and K_CO_, respectively) are generally preserved in asthma and deficient in COPD [2, 4], they might vary widely among ACO cases depending on the proportion of each component of asthma or COPD. However, among patients with ACO, the distributions of D_LCO_ and K_CO_, as well as impairing factors for them, have not been investigated.

There is broad agreement that patients with ACO experience exacerbations more frequently than those with asthma or COPD alone [1, 3]. With regard to COPD, exacerbations are an ideal target for risk-stratified treatment to lead to a higher health-related quality of life, longer life, and lower healthcare cost [5]. Recent evidence indicates that impairment in D_LCO_ is a strong biomarker for predicting the risk of severe exacerbation in COPD [6]. It is assumed that D_LCO_ or K_CO_ can be applied as a risk factor for exacerbations in ACO; however, there has been no study evaluating this issue. Thus, assessing the influence of D_LCO_ and K_CO_ on ACO exacerbations could be of great benefit for improving health management for such patients.

Based on these considerations, the present study was conducted to evaluate the distributions of gas diffusion and transfer of the lung, explore factors that influence them, and assess their clinical usefulness in predicting future exacerbations among ACO patients.

## Methods

### Study population

The present study was conducted as an observational cohort study through a review of medical records at National Hospital Organization Fukuoka National Hospital. The cohort consisted of 94 ACO patients aged ≥ 40 years who had an assessment of D_LCO_ and K_CO_ from June 1, 2017, to May 31, 2020, with complete information on all relevant covariates. ACO was defined as the presence of three major criteria: (i) persistent airflow limitation, that is, post-bronchodilator forced expiratory volume in 1 s to forced vital capacity (post-BD FEV_1_/FVC) < 70%; (ii) at least one feature associated with COPD; and (iii) one or more asthmatic features.

The COPD-like features were composed of (a) a smoking history of > 10 pack-years and (b) pulmonary emphysema. The asthmatic features were as follows: (a) high values of fractional exhaled nitric oxide (FeNO) (> 35 parts per billion (ppb)), (b) bronchodilator reversibility (≥ 12% and ≥ 200 ml reversibility in post-BD FEV_1_), (c) an eosinophilic component (blood eosinophil levels > 300/µl and/or > 5%), and (d) positive levels for total immunoglobulin E (IgE) (> 170 IU/ml) and/or IgE specific to perennial inhalant antigens (> class 2). Subjects with ≥ 3 asthmatic features were defined as “highly asthmatic” ones. The distribution of and affecting factors for D_LCO_ per predicted (D_LCO_ % pred) as well as K_CO_ per predicted (K_CO_ % pred) were evaluated as a cross-sectional analysis using the total cohort. After excluding 4 patients with no follow-up data, the remaining 90 patients were also recruited in the prospective research (the prospective cohort) in order to investigate the association of D_LCO_ % pred and K_CO_ % pred with exacerbations of ACO.

### Assessment of diffusing capacity and transfer coefficient of the lung

D_LCO_ and K_CO_ were measured via the single-breath method using CHESTAC-8900 (Chest Inc., Tokyo, Japan) in accordance with the American Thoracic Society / European Respiratory Society (ATS/ERS) guidelines [7]. D_LCO_ % pred was calculated using the predicted D_LCO_ value for a person of the same age, gender, and body surface area [8]. Likewise, K_CO_ % pred was estimated based on the age-dependent prediction equation for K_CO_ [8]. In accordance with the clinical review article [4], low D_LCO_ and low K_CO_ were defined as D_LCO_ % pred < 80% and K_CO_ % pred < 80%, respectively. When dividing the prospective cohort into three groups based on the tertile distribution of D_LCO_ % pred or K_CO_ % pred, the cut-off values were as follows: lowest, ≤ 82.0%; middle, 82.1–108.72%; and highest, ≥ 108.73% for D_LCO_ % pred; lowest, ≤ 79.9%; middle, 80.0–97.9%; and highest, ≥ 98.0% for K_CO_ % pred. To assess the robustness of the outcomes, we also estimated D_LCO_ % pred and K_CO_ % pred using another reference formula, namely the lambda, mu, and sigma method employed by the ERS Global Lung Function Initiative (GLI) Task Force [9].

### Clinical evaluations

For each case, respiratory physicians reviewed the patient’s medical records and assessed the demographic and clinical characteristics: age, gender, height, weight, smoking exposure, medical history, blood laboratory findings, spirometry, FeNO, computed tomography (CT) scans of the chest, and medical treatment for ACO, including systemic corticosteroid and inhaled long-acting bronchodilators (LABDs), namely long-acting β_2_ agonists and long-acting muscarinic antagonists, and inhaled corticosteroid (ICSs). CT scanning was executed with a minimal slice thickness of 1–5 mm. The proportion of subjects with systemic corticosteroid use was 8.5% among the entire cohort. Body mass index (BMI; kg/m^2^) was calculated as weight divided by squared height. Overweight and underweight were defined as BMI ≥ 25.0 kg/m^2^ and BMI < 18.5 kg/m^2^, respectively. Spirometry was performed fifteen minutes after bronchodilator administration, in line with the guidelines of the Japanese Respiratory Society [10], using a CHESTAC-8900 spirometer (Chest MI, Tokyo, Japan). According to the Global Initiative for Chronic Obstructive Lung Disease (GOLD) criteria [11], the severity of ACO was defined using the predicted FEV_1_ value for a person of the same age, gender, and height using the equation for the Japanese population [12] as follows: mild, FEV_1_ ≥ 80% of predicted; moderate, 50% ≤ FEV_1_ < 80% of predicted; severe and very severe, FEV_1_ < 50% of predicted. Based on the official statement of ATS/ERS [13], an exacerbation of ACO was defined as worsening symptoms that require treatment with oral/systemic corticosteroids and/or antibiotics for at least three days. Together with a radiologist, respiratory physicians interpreted the chest CT images for each case and scrutinised them for the presence of emphysema.

### Statistical analysis

SAS University Edition software version 9.4 (SAS Institute, Cary, NC, USA) was used to perform all statistical analyses. A two-sided *P* < 0.05 was considered to indicate statistical significance. The effects of the potential risk factors on the prevalence of low D_LCO_ and low K_CO_ were estimated as odds ratios (ORs) with 95% confidence intervals (95% CIs) in a multivariable-adjusted logistic regression model, wherein adjustment was made for age, gender, BMI, FEV_1_, use of LABDs and ICSs, features associated with COPD (a smoking history of > 10 pack-years, and emphysema), and those associated with asthma (high FeNO, bronchial reversibility, an eosinophilic component, and a positive total IgE and/or IgE specific to perennial inhalant antigens). Kaplan–Meier curves were constructed to show the exacerbation-free rate over the one-year period. Log-rank testing was performed to study the influence of low D_LCO_ and low K_CO_ on the cumulative incidence of exacerbations. The hazard ratios (HRs) with their 95% CIs according to the levels of D_LCO_ % pred or K_CO_ % pred for the development of exacerbation were estimated using a Cox proportional hazards model adjusted for history of exacerbations in the previous year in addition to all aforementioned potential confounders. The same model was used to assess the linear trends in the risk of exacerbation across the tertile classification of D_LCO_ % pred or K_CO_ % pred.

Sensitivity analyses were performed using the patients without systemic corticosteroid use. Another analysis was conducted with GLI-based D_LCO_ % pred and K_CO_ % pred.

### Ethical considerations

The study was approved by the National Hospital Organization Fukuoka National Hospital Institutional Review Board for Clinical Research (#F3-3).

## Results

### Distributions of DLCO % pred and KCO % pred

Table 1 lists the demographic and clinical characteristics among the total cohort. The majority of the individuals were male (81.9%), with smoking exposure of ≥ 10 pack-years (92.6%) and with increased levels of total IgE and/or IgE specific to perennial inhalant antigens (79.8%). Nearly half of the total cohort (42.6%) experienced exacerbations in the previous year. The distribution of severity was as follows: mild, 28.7%; moderate, 42.6%; and severe/very severe, 28.7%. The prevalence of highly asthmatic cases was higher among ICS users than among nonusers, although not statistically different (37.5% vs. 20.0%, respectively; *P* = 0.09).

**Table 1:** Mean values or frequencies of demographic and clinical characteristics

Variables	Mean values (standard deviation), median values (interquartile range), or frequencies
Male gender (%)	81.9
Age (years)	69.8 (9.1)
Body surface area (m^2^)	1.66 (0.19)
Body mass index (kg/m^2^)	22.7 (3.4)
Smoking history (pack-year)	39.0 (20.0–50.0)
Smoking history ≥ 10 pack-years (%)	92.6
Emphysema (%)	68.1
Blood eosinophil levels (%)	5.2 (2.8–8.9)
Blood eosinophil counts (/µl)	351 (150–538)
Eosinophilic component (%)	59.6
FeNO (ppb)	31.5 (19.0–59.0)
High FeNO (%)	46.8
Total IgE levels (IU/ml)	406 (119–868)
Positive levels for total IgE and/or IgE specific to perennial inhalant antigens (%)	79.8
Bronchial reversibility (%)	19.1
FEV_1_ (l)	1.64 (0.67)
FEV_1_ per predicted	
Disease severity	
Mild (%)	28.7
Moderate (%)	42.6
Severe/very severe (%)	28.7
≥ 1 exacerbation in the previous year (%)	42.6
Inhaled long-acting bronchodilator use (%)	72.3
Inhaled corticosteroid use (%)	68.1
Systemic corticosteroid use (%)	8.5

Age, body surface area, body mass index, and FEV_1_ are given as the mean with standard deviations. Smoking history, blood eosinophil levels and counts, FeNO, and total IgE levels are shown as the median with an interquartile range because of their skewed distributions. Other variables are given as the number of cases and percentages. The eosinophilic component was defined as blood eosinophil ≥ 5% and/or ≥ 300/µl. High FeNO was defined as > 35 ppb. Bronchial reversibility was defined as ≥ 12% and ≥ 200 ml reversibility in post-bronchodilator FEV_1_. Positive levels for total IgE were defined as > 170 IU/ml. Inhaled long-acting bronchodilator use referred to the use of long-acting β_2_ agonists and/or long-acting muscarinic antagonists

BMI, body mass index; FeNO, fractional exhaled nitric oxide; ppb, parts per billion; IgE, immunoglobulin E; FEV_1_, forced expiratory volume in 1 s

**Fig. 1:**
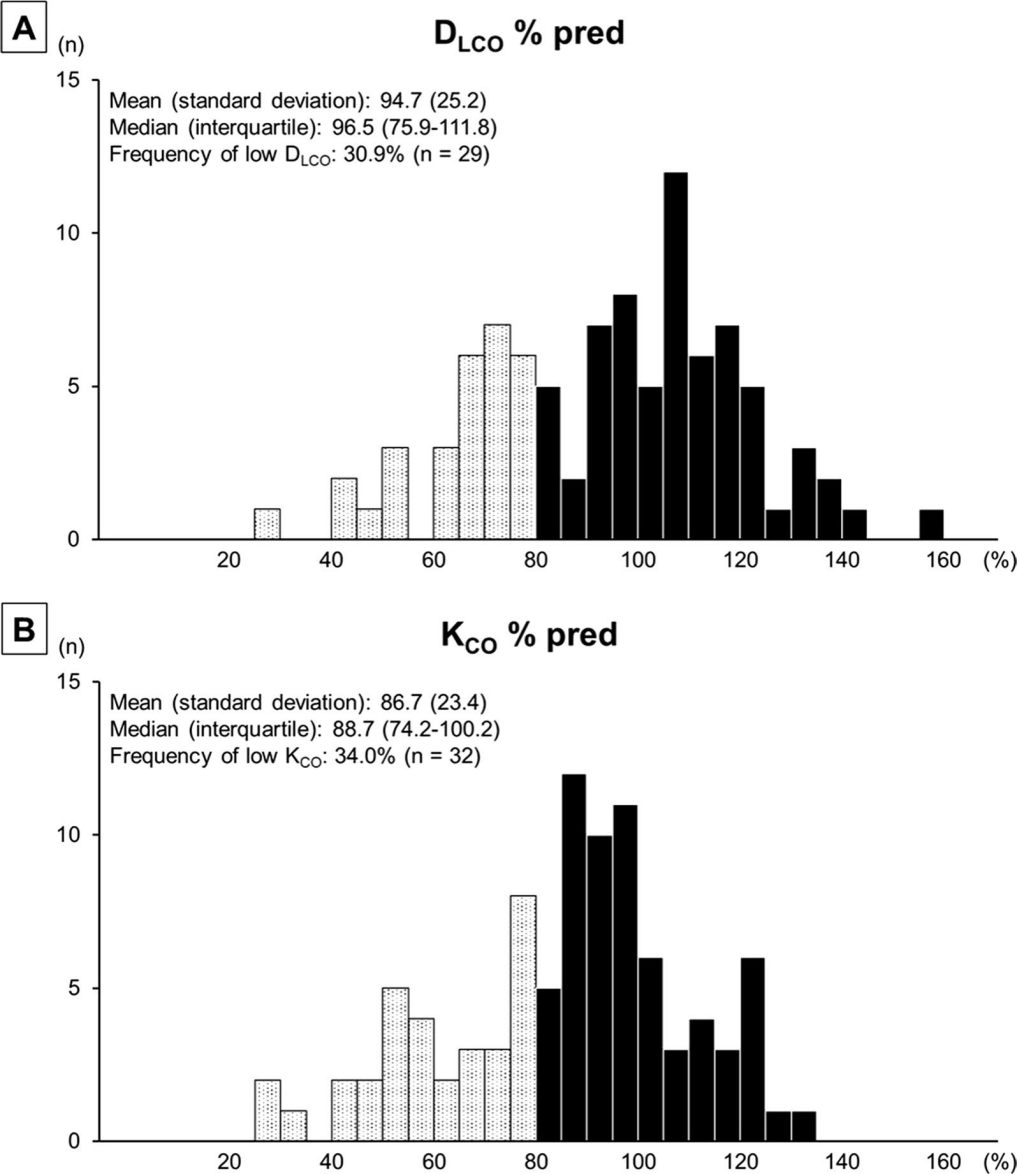
Distribution of diffusing capacity (**A**) and transfer coefficient (**B**) of the lung per predicted among the total cohort. D_LCO_ % pred, diffusing capacity for carbon monoxide per predicted; K_CO_ % pred, transfer coefficient for carbon monoxide per predicted

As in Fig. 1A, B, both D_LCO_ % pred and K_CO_ % pred showed approximately normal distributions. The prevalence of low D_LCO_ among the subjects was 30.9%. Decreases in K_CO_ were also observed in about one-third of the subjects (34.0%).

### Factors affecting DLCO % pred and KCO % pred

FEV_1_ and the use of ICSs were inversely associated with low D_LCO_ (multivariable-adjusted OR = 0.29 (95% CI 0.10–0.76) for a 1-L increase in FEV_1_ and 0.07 (95% CI 0.01–0.59) for ICS use, respectively) (Table 2), whereas neither of them independently affected the prevalence of low K_CO_. On the other hand, LABD use was associated with neither low D_LCO_ nor low K_CO_. There was a negative relationship between BMI and low K_CO_ (multivariable-adjusted OR = 0.73 (95% CI 0.57–0.90)). The prevalence of low K_CO_ was significantly higher in subjects with emphysema than in those without (multivariable-adjusted OR = 7.37 (95% CI 1.81–40.3)) (Table 3). While the prevalence of low D_LCO_ was not statistically different between subjects with/without emphysema (multivariable-adjusted OR = 1.32 (95% CI 0.39–4.77)) (Table 2). Regarding other variables, there was no significant relationship to either low D_LCO_ or low K_CO_.

**Table 2:** Multivariable-adjusted odds ratios of potential risk factors for low D_LCO_

Variables	Number of events/cases (%)		Multivariable-adjusted OR (95% CI)	*P* value
	Exposure group	Reference group		
Male gender	21/77 (27.3%)	8/17 (47.3%)	0.90 (0.18–4.40)	0.89
Age (per 10-year increase)	N/A	N/A	0.83 (0.41–1.66)	0.60
BMI (per 1 kg/m^2^ increase)	N/A	N/A	1.03 (0.86–1.22)	0.76
FEV_1_ (per 1 L increase)	N/A	N/A	0.29 (0.10–0.76)	0.02
Inhaled long-acting bronchodilator use	22/68 (32.4%)	7/26 (26.9%)	8.42 (0.93–108)	0.07
ICS use	17/64 (26.6%)	12/30 (40.0%)	0.07 (0.01–0.59)	0.02
*COPD-like features*				
Smoking history ≥ 10 pack-years	28/87 (32.2%)	1/7 (14.3%)	3.32 (0.34–80.7)	0.36
Emphysema	22/64 (34.4%)	7/30 (23.3%)	1.32 (0.39–4.77)	0.66
*Asthmatic features*				
Eosinophilic component	18/56 (32.1%)	11/38 (28.9%)	1.65 (0.51–5.71)	0.41
High FeNO	11/44 (25.0%)	18/50 (36.0%)	0.51 (0.15–1.61)	0.26
Positive levels for total IgE and/or IgE specific to perennial inhalant antigens	21/75 (28.0%)	8/19 (42.1%)	0.51 (0.12–2.06)	0.34
Bronchial reversibility	5/18 (27.8%)	24/76 (31.6%)	1.08 (0.24–4.20)	0.92

Adjustment was made for age, gender, BMI, FEV_1_, long-acting bronchodilator use, ICS use, a smoking history of > 10 pack-years, emphysema, high FeNO, bronchial reversibility, an eosinophilic component, and positive levels for total IgE and/or IgE specific to perennial inhalant antigens. The eosinophilic component was defined as blood eosinophil ≥ 5% and/or ≥ 300/µl. High FeNO was defined as ≥ 35 parts per billion. Bronchial reversibility was defined as ≥ 12% and ≥ 200 ml reversibility in post-bronchodilator FEV_1_. Positive levels for total IgE were defined as > 170 IU/ml

OR, odds ratio; 95% CI, 95% confidence interval; BMI, body mass index; FEV_1_, forced expiratory volume in 1 s; ICS, inhaled corticosteroid; COPD, chronic obstructive pulmonary disease; N/A, not applicable

**Table 3:** Multivariable-adjusted odds ratios of potential risk factors for low K_CO_

Variables	Number of events/cases (%)		Multivariable-adjusted OR (95% CI)	*P* value
	Exposure group	Reference group		
Male gender	28/77 (36.4%)	4/17 (23.5%)	1.77 (0.26–13.5)	0.56
Age (per 10-year increase)	N/A	N/A	1.12 (0.52–2.41)	0.77
BMI (per 1 kg/m^2^ increase)	N/A	N/A	0.73 (0.57–0.90)	< 0.01
FEV_1_ (per 1 L increase)	N/A	N/A	1.67 (0.62–4.72)	0.32
Inhaled long-acting bronchodilator use	24/68 (35.3%)	8/26 (30.8%)	2.22 (0.25–23.1)	0.48
ICS use	21/64 (32.8%)	11/30 (36.7%)	0.74 (0.08–6.52)	0.79
*COPD-like features*				
Smoking history ≥ 10 pack-years	30/87 (34.5%)	2/7 (28.6%)	1.84 (0.20–21.9)	0.60
Emphysema	29/64 (45.3%)	3/30 (10.0%)	7.37 (1.81–40.3)	< 0.01
*Asthmatic features*				
Eosinophilic component	21/56 (37.5%)	11/38 (28.9%)	2.20 (0.66–8.14)	0.21
High FeNO	12/44 (27.3%)	20/50 (40.0%)	0.36 (0.09–1.23)	0.11
Positive levels for total IgE and/or IgE specific to perennial inhalant antigens	21/75 (28.0%)	11/19 (57.9%)	0.42 (0.09–1.70)	0.23
Bronchial reversibility	5/18 (27.8%)	27/76 (35.5%)	0.47 (0.09–2.15)	0.35

Adjustment was made for age, gender, BMI, FEV_1_, long-acting bronchodilator use, ICS use, a smoking history of > 10 pack-years, emphysema, high FeNO, bronchial reversibility, an eosinophilic component, and positive levels for total IgE and/or IgE specific to perennial inhalant antigens. The eosinophilic component was defined as blood eosinophil ≥ 5% and/or ≥ 300/µl. High FeNO was defined as ≥ 35 parts per billion. Bronchial reversibility was defined as ≥ 12% and ≥ 200 ml reversibility in post-bronchodilator FEV_1_. Positive levels for total IgE were defined as > 170 IU/ml

OR, odds ratio; 95% CI, 95% confidence interval; BMI, body mass index; FEV_1_, forced expiratory volume in 1 s; ICS, inhaled corticosteroid; COPD, chronic obstructive pulmonary disease; N/A, not applicable

### Association of DLCO % pred and KCO % pred with exacerbations of ACO

**Fig. 2:**
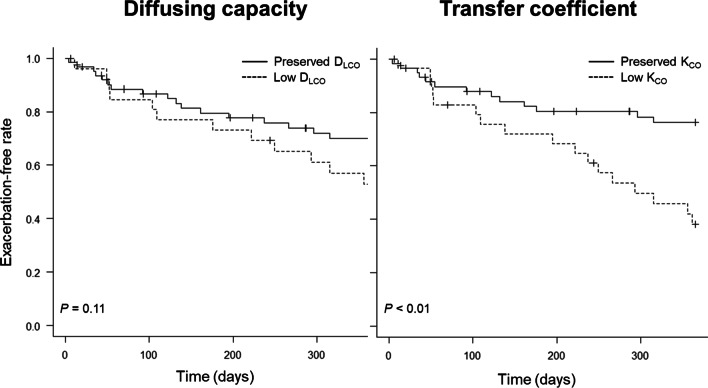
The exacerbation-free rate in one year according to the levels of diffusion capacity or transfer coefficient of the lung. Low and preserved D_LCO_ were defined as D_LCO_ % pred < 80% and ≥ 80%, respectively. In the same manner, low and preserved K_CO_ indicated K_CO_ % pred < 80% and ≥ 80%, respectively

**Fig. 3:**
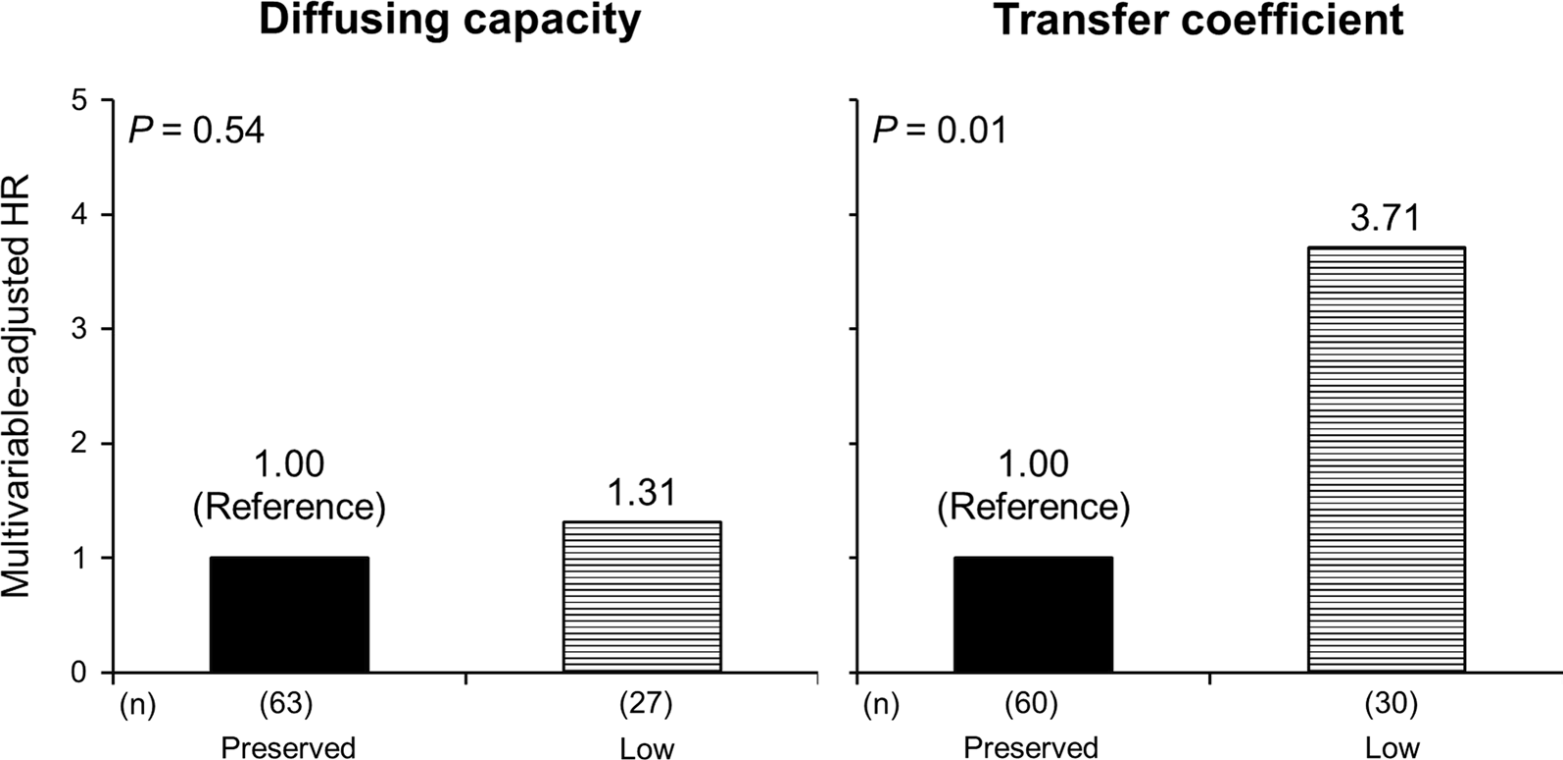
The multivariable-adjusted hazard ratios for exacerbation by the levels of diffusion capacity or transfer coefficient of the lung. HR, hazard ratio. With regard to diffusing capacity, the preserved and low groups consisted of subjects with D_LCO_ % pred ≥ 80% and < 80%, respectively. Similarly, the preserved-K_CO_ and low-K_CO_ group indicated subjects with K_CO_ % pred < 80% and ≥ 80%, respectively

**Fig. 4:**
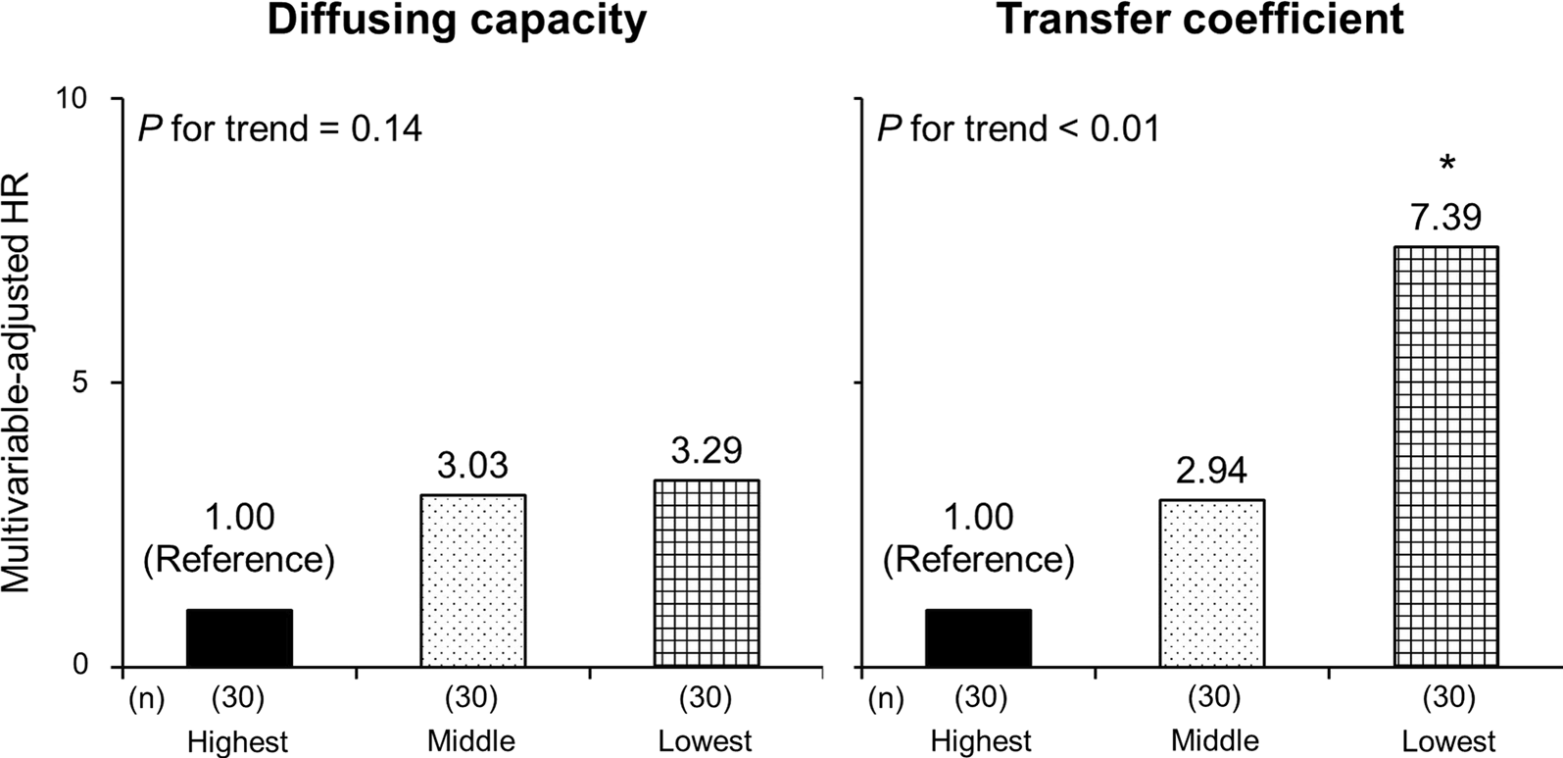
The multivariable-adjusted hazard ratios for exacerbation according to the tertile of diffusion capacity or transfer coefficient of the lung. HR, hazard ratio. *P < 0.01 versus the reference group. With regard to diffusing capacity, the cut-off values for D_LCO_ % pred are indicated as follows: highest, ≥ 108.73%; middle, 82.1–108.72%; and lowest, ≤ 82.0%. Similarly, the cut-offs for K_CO_ % pred were ≥ 98.0% for the highest, 80.0–97.9% for the middle, and ≤ 79.9% for the lowest tertile group

In the prospective cohort, 30 individuals (33.3% of the cohort) experienced at least one event of ACO exacerbation within one year. The incidence rate of ACO exacerbations over one year was significantly higher in the low-K_CO_ group than in the other (*P* = 0.002), while there was no significant difference between the low- and preserved-D_LCO_ groups (*P* = 0.11) (Fig. 2). The results were substantially similar after adjustment for potential confounders: there was a significant increase in HR in the low-K_CO_ group as compared to the preserved-K_CO_ group (HR = 3.71 (95% CI 1.32–10.4)), whereas there was not for the D_LCO_ groups (HR = 1.31 (95% CI 0.55–3.11)) (Fig. 3). As shown in Fig. 4, there was a significant linear trend between K_CO_ % pred and the incidence of exacerbation (*P* = 0.003 for the trend). Compared with the highest tertile group, the multivariable-adjusted HR for ACO exacerbations was significantly higher in the lowest tertile group (HR = 7.39 (95% CI 1.94–28.2)). Meanwhile, the association of D_LCO_ % pred with exacerbations failed to reach statistical significance (*P* = 0.14 for the trend). Broadly similar results were obtained in the analysis among patients without systemic corticosteroid use (Additional file 1: e-Figs. 1–3) and the analysis using ERS GLI reference equations (Additional file 1: e-Figs. 4–6).

## Discussion

The present study revealed the prevalence of both low D_LCO_ and low K_CO_ in about one-third of the patients with ACO. There were inverse associations of FEV_1_ and the use of ICSs with low D_LCO_, while low K_CO_ was associated positively with the presence of emphysema and negatively with BMI. Our study also showed that impaired K_CO_ % pred was a significant risk factor for exacerbation of ACO. To the best of our knowledge, this is the first study to evaluate the distributions of D_LCO_ % pred and K_CO_ % pred, investigate risk factors, and estimate the amount of their impacts on exacerbations in ACO patients.

In the present research, low K_CO_ was an independent risk factor for severe exacerbations of ACO, while low D_LCO_ was not. As to COPD, the most robust predictors of future exacerbation had been considered to be a reduction in FEV_1_ and a history of exacerbations in the previous year [5, 14–17]. In addition, recent large-scale cohort studies demonstrated that impaired diffusing capacity was also strongly associated with increased rates of exacerbation among subjects with COPD [6, 18]. As compared to D_LCO_, K_CO_ better reflects smoking-related injury of the lung in asthma as well as COPD patients [19]; in the current research, K_CO_ is speculated to be a stronger risk factor than D_LCO_ for exacerbations in ACO. Since diffusion capacity and gas transfer correlate with exercise capacity in COPD patients [20], deficits in K_CO_ can lead to exercise inactivity, physical deconditioning, and disease advances. Taking into account the multivariable adjustment made in the current research methods, K_CO_ might be a more appropriate biomarker than the previously established ones (e.g., FEV_1_ and exacerbation history) to detect the frequent-exacerbation phenotype of ACO, although further investigation is needed.

It has been well known that COPD is commonly accompanied by a reduction in diffusing capacity and transfer coefficient, while asthma is not [2]. In COPD, the pathophysiologic mechanisms underlying the deterioration of D_LCO_ and K_CO_ are due to alveolar destruction, namely emphysema, and alveolar microvascular damage preceding emphysematous changes [21]. On the other hand, regarding asthma, both D_LCO_ % pred and K_CO_ % pred are preserved or even increased to some extent because of the redistribution of pulmonary blood flow [22]. In our study, lung diffusion impairment was observed in only about one-third of the ACO cases; the pathophysiologic aspects of asthma may have compensated for the decline in gas diffusion and transfer due to COPD.

An increase in FEV_1_ was associated with a decrease in the prevalence of low D_LCO_ in the current study. This is considered to be because D_LCO_, not K_CO_, is in proportion to lung volume, since D_LCO_ is the product of K_CO_ and alveolar volume at measurement. The relationship between ICS use and D_LCO_ was compatible with previous reports; concomitant use of ICSs and LABDs has favourable effects on diffusing capacity, while LABD use alone does not [23, 24]. The anti-inflammatory actions of ICSs might have prevented the progression of airflow limitation and lung hyperinflation, resulting in optimisation of the functionally available alveolar volume and the restoration of D_LCO_ [23]. Another possible explanation is that ICS-prescribed patients were more asthmatic and less prone to develop deficits in D_LCO_ than the others, although there was no significant difference in the frequency of highly asthmatic subjects between ICS users and nonusers in the current results.

In our research, emphysema was an independent risk factor for low K_CO_. With regard to the effects of pulmonary emphysema on K_CO_ and D_LCO_ in the present study, similar results were observed among the cohort of patients with asthma or COPD [20], reinforcing the evidence that K_CO_ is more sensitive than D_LCO_ to detect the development of emphysema [25]. Considering the statistical significance after adjustment for alveolar volume substituted with FEV_1_, a high probability of K_CO_ impairment in cases with emphysema might have been due to pulmonary microvascular remodelling as an early-stage change in COPD rather than emphysematous destruction per se [21]. It was also found that a higher BMI was inversely associated with the prevalence of not low D_LCO_ but low K_CO_, which was in accord with previous studies [26]. The association between BMI and K_CO_ was likely to be attributed to an increase in pulmonary capillary blood volume among the obese [26, 27]. Meanwhile, in obesity, an increase in abdominal pressure and mechanical constraint placed on the chest wall by fat accumulation leads to low lung volumes and, consequently, attenuates the impact of elevated K_CO_ on D_LCO_ [26, 28].

The strengths of our study were the highly accurate diagnosis of ACO using various objective measurements to assess the likelihood of asthma and COPD, the uniformity in measurements of D_LCO_ and K_CO_ by virtue of single-centre outcomes, the use of regression models adjusting for multiple confounders to evaluate the independent effects of D_LCO_ and K_CO_, and the prospective research design to minimise the potential of reverse causation.

However, some potential limitations should be noted. First, both D_LCO_ and K_CO_ values were based on a single measurement. This may cause misclassification of the levels of potential for gas exchange, which could have weakened the associations found in the present study, biasing the results toward a null hypothesis. Second, the present outcomes might lack external validity and generalisability due to the study design as single-centre analyses, although the characteristics of the study population were substantially comparable to those of other multicentre cohorts [29, 30]. Third, systemic corticosteroid therapy might have affected the current results. However, among the study cohort, the proportion of subjects with systemic corticosteroid exposure was only 8.5%. Furthermore, a sensitivity analysis excluding the patients with systemic corticosteroid use showed results consistent with those in the primary analysis. Thus, this limitation may not be critical.

Fourth, we did not have access to the data on air-trapping measurement due to the retrospective nature of the study. Fifth, we were unable to adjust the confounding effects of active smoking due to a lack of data concerning the present status of smoking or blood carboxyhaemoglobin concentration. Instead, through the present analysis, the number of pack-years of cigarette smoking was adjusted, along with other covariates. Sixth, the reference equations for D_LCO_ and K_CO_ applied in the main analyses might not have been applicable to the cohort subjects. However, a sensitivity analysis using the GLI reference equation provided outcomes similar to those in the primary analysis; this limitation would not have changed our conclusion. Seventh, we defined persistent airflow limitation as post-BD FEV_1_/FVC < 70%, not the lower limit of normal, based on findings from a large-scale general-population cohort study [31] and the official report of the Global Initiative for Chronic Obstructive Lung Disease [32].

Eighth, the possibility of overlooking early emphysema could not be denied, although ACO and COPD were assessed and diagnosed based on smoking history and the existence of persistent airflow limitation, as well as CT images; early emphysema without COPD was therefore ruled out in the study population [33]. Ninth, we did not have access to a tool for radiological assessment of the severity of pulmonary emphysema and lung volume. Instead of the severity of emphysema and lung volume, the value of FEV_1_ was adjusted in the analyses. Lastly, there was a possibility of confounding effects of inhaler medications on the present results. However, it has been reported that an LABD was unable to contribute to significant improvements in either D_LCO_ or K_CO_ [24]. Combined use of an ICS and LABD could have been favourable for both the diffusion capacity and exacerbation rate [23] and might have affected the associations between D_LCO_ or K_CO_ and exacerbation events. However, the present outcomes were demonstrated after adjustment for inhaler use, suggesting that this potential limitation may not have altered our conclusions.

## Conclusions

About one-third of the cases of ACO presented with low D_LCO_, which was associated with decreased FEV_1_. The prevalence of low K_CO_ was comparable to that of impaired D_LCO_ and was higher in the subjects with emphysema. Additionally, low K_CO_ was an independent risk factor for ACO exacerbations, and there was a linear trend in the risk of exacerbation across the level of K_CO_ % pred. Since K_CO_ is superior to DL_CO_ in reflecting initial development of microvascular or parenchymal damage of the lung in ACO, K_CO_ may be more useful than D_LCO_ for predicting the future risk of exacerbations. In clinical practice, ACO patients with low K_CO_ should be carefully monitored due to their high potential for exacerbations. Further research is warranted to clarify whether K_CO_ is a biomarker for mortality in subjects with ACO.

## Supplementary Information

**Additional file 1.** Supplementary materials (e-Figs. 1-6).

## Data Availability

The datasets used and/or analysed during the current study are available from the corresponding author on reasonable request.

## Abbreviations

COPD: Chronic obstructive pulmonary disease
ACO: Asthma–COPD overlap
D_LCO_: Diffusing capacity of the lung for carbon monoxide
K_CO_: Transfer coefficient of the lung for carbon monoxide
BD: Bronchodilator
FEV_1_/FVC: Forced expiratory volume in 1 s to forced vital capacity
FeNO: Fractional exhaled nitric oxide
ppb: Parts per billion
ATS/ERS: American Thoracic Society/European Respiratory Society
IgE: Immunoglobulin E
% pred: Percent predicted
GLI: Global Lung Function Initiative
CT: Computed tomography
LABD: Long-acting bronchodilator
ICS: Inhaled corticosteroid
BMI: Body mass index
GOLD: Global Initiative for Chronic Obstructive Lung Disease
HR: Hazard ratio
OR: Odds ratio
95% CIs: 95% Confidence intervals
