# Supplementary material for: The association between transfer coefficient of the lung and the risk of exacerbation in asthma-COPD overlap: an observational cohort study
Source: BMC Pulm Med. 2022 Jan 12;22:22. doi: 10.1186/s12890-021-01815-w (PMC8753934; doi:10.1186/s12890-021-01815-w)
Supplement: Supplementary file 1 — Additional file 1. Supplementary materials (e-Figs. 1-6). [file 12890_2021_1815_MOESM1_ESM.docx]

**Additional file**

**e-Figure 1.** The exacerbation-free rate in one year according to the levels of diffusion capacity or transfer coefficient of the lung among the patients without systemic corticosteroid use.


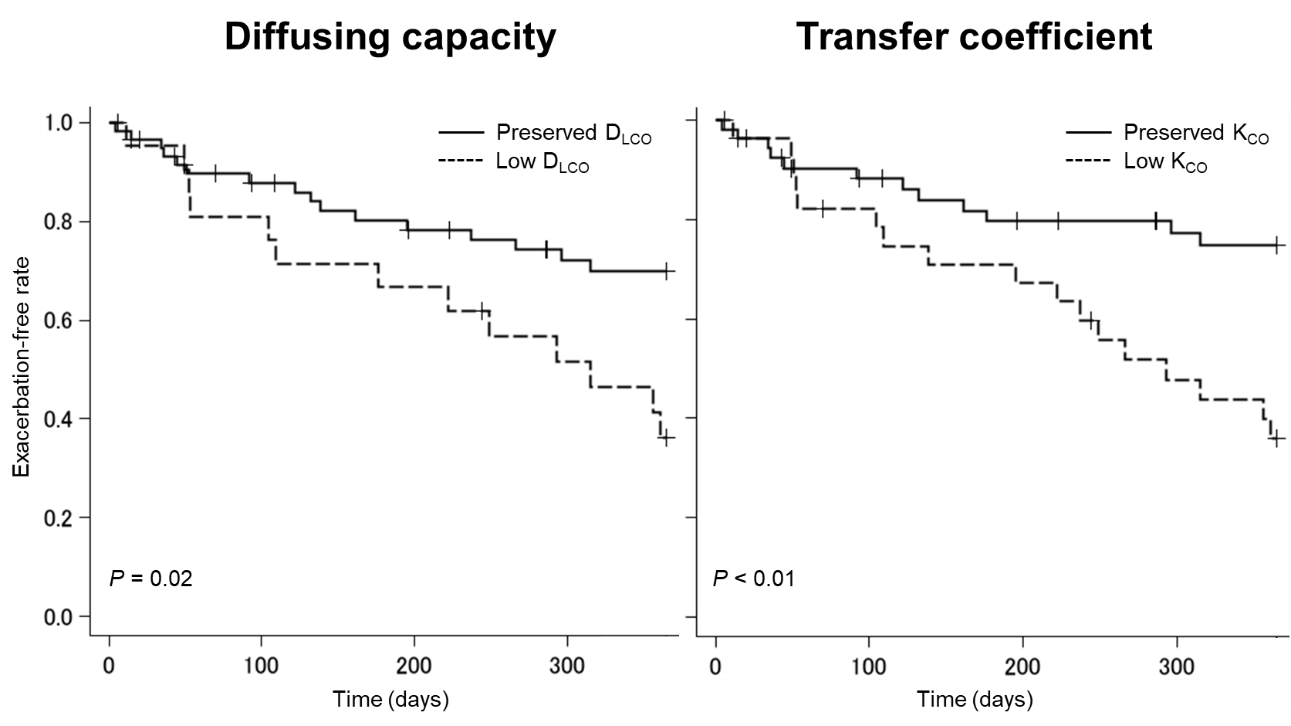


GLI, Global Lung Function Initiative.

Low and preserved D_LCO_ were defined as D_LCO_ % pred < 80% and ≥ 80%, respectively. In the same manner, low and preserved K_CO_ indicated K_CO_ % pred < 80% and ≥ 80%, respectively.

**e-Figure 2.** The multivariable-adjusted hazard ratios for exacerbation by the levels of diffusion capacity or transfer coefficient of the lung among the patients without systemic corticosteroid use.


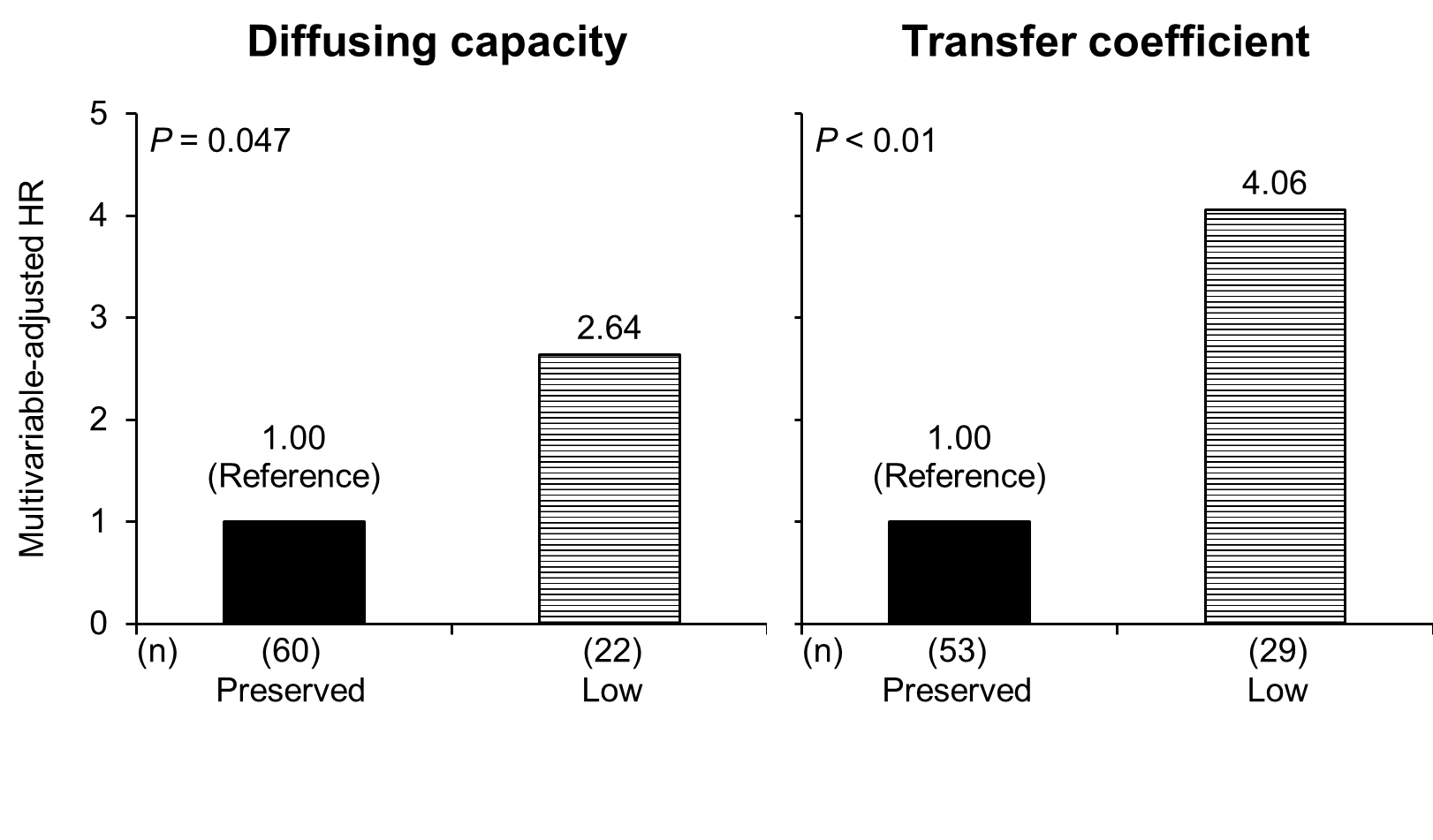


GLI, Global Lung Function Initiative; HR, hazard ratio.

With regard to diffusing capacity, the preserved and low groups consisted of subjects with D_LCO_ % pred ≥ 80% and < 80%, respectively. Similarly, the preserved-K_CO_ and low-K_CO_ group indicated subjects with K_CO_ % pred < 80% and ≥ 80%, respectively.

**e-Figure 3.** The multivariable-adjusted hazard ratios for exacerbation according to the tertile of diffusion capacity or transfer coefficient of the lung among the patients without systemic corticosteroid use.


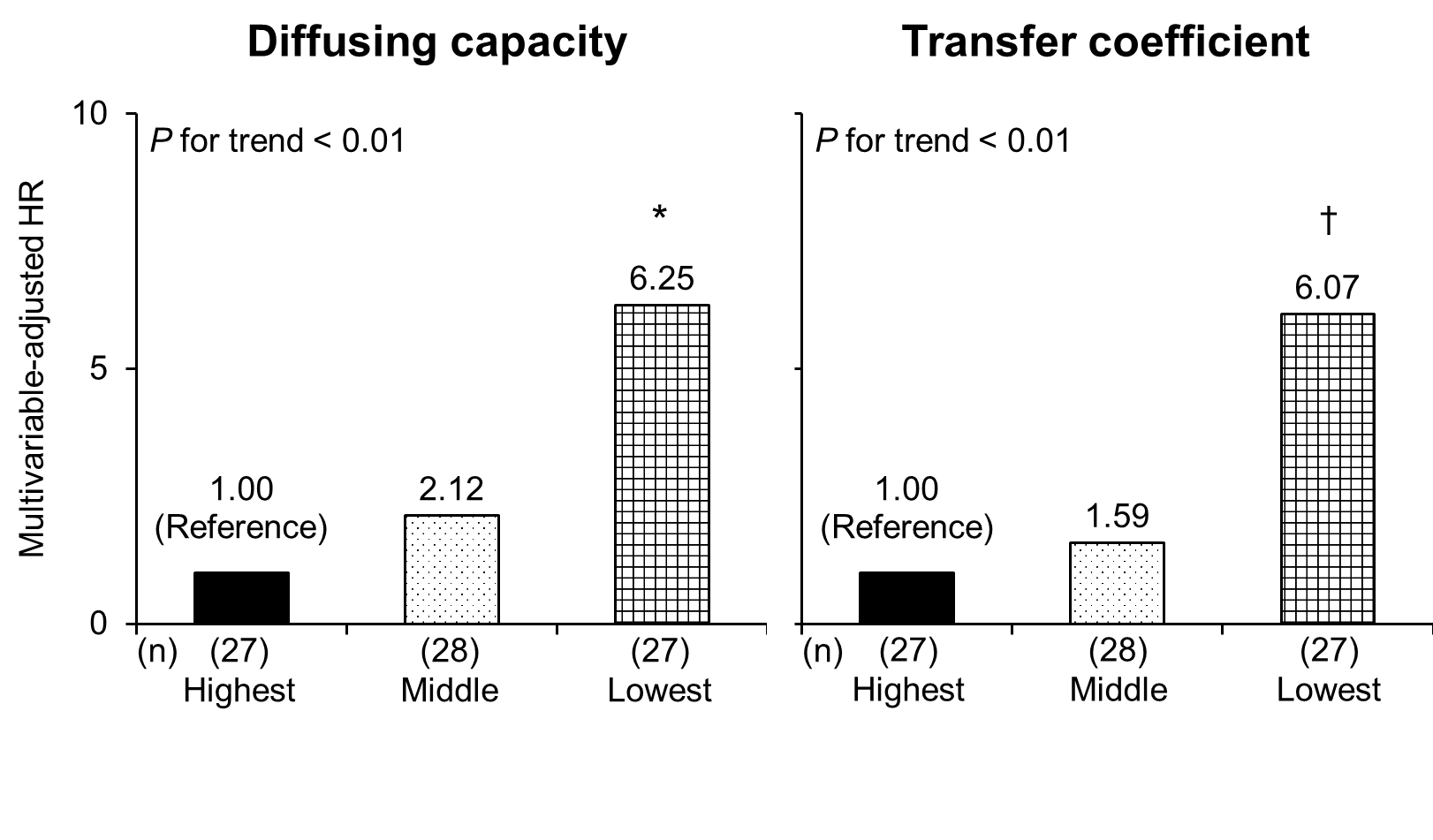


GLI, Global Lung Function Initiative; HR, hazard ratio.

^*^*P* < 0.05 versus the reference group.

^†^*P* < 0.01 versus the reference group.

With regard to diffusing capacity, the cut-off values for D_LCO_ % pred are indicated as follows: highest, ≥ 109.0%; middle, 89.0–108.9%; and lowest, ≤ 88.9%. Similarly, the cut-offs for K_CO_ % pred were ≥ 97.50% for the highest, 78.60–97.49% for the middle, and ≤ 78.59% for the lowest tertile group.

**e-Figure 4.** The exacerbation-free rate in one year according to the levels of GLI-based diffusion capacity or transfer coefficient of the lung.


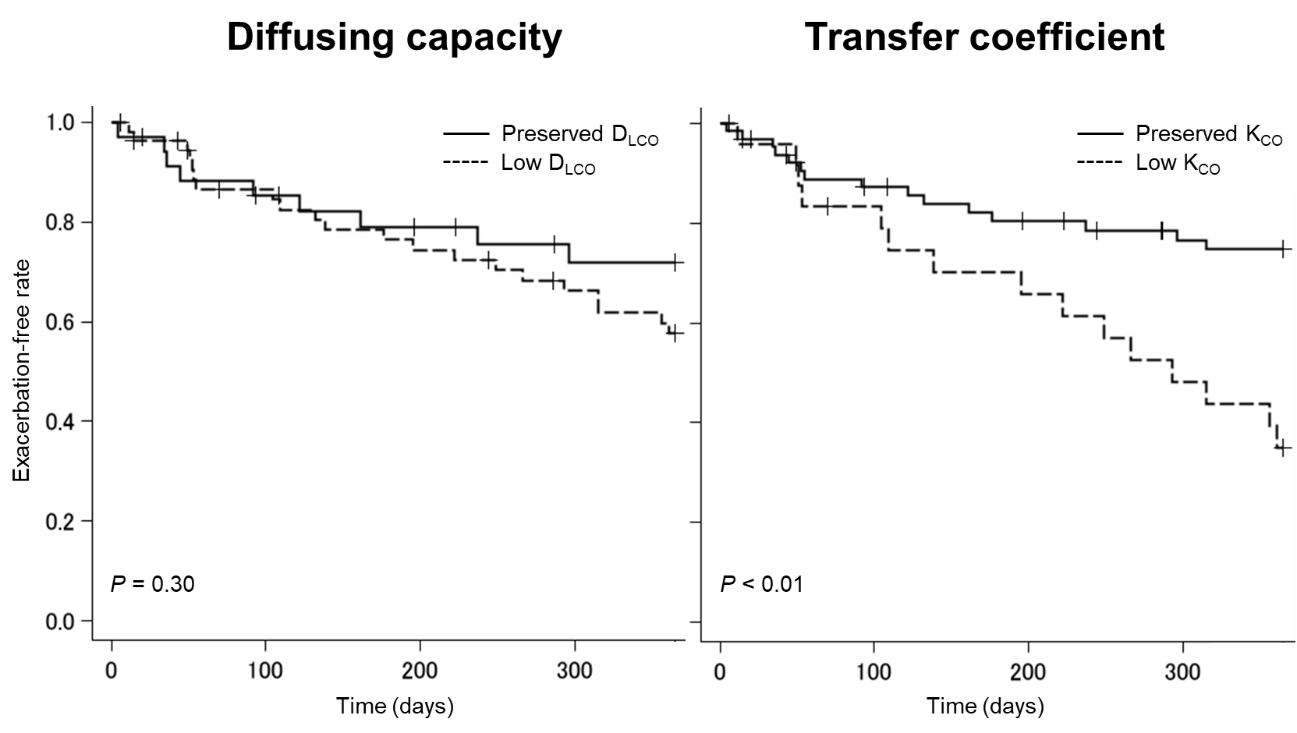


GLI, Global Lung Function Initiative.

Low and preserved D_LCO_ were defined as D_LCO_ % pred < 80% and ≥ 80%, respectively. In the same manner, low and preserved K_CO_ indicated K_CO_ % pred < 80% and ≥ 80%, respectively.

**e-Figure 5.** The multivariable-adjusted hazard ratios for exacerbation by the levels of GLI-based diffusion capacity or transfer coefficient of the lung.


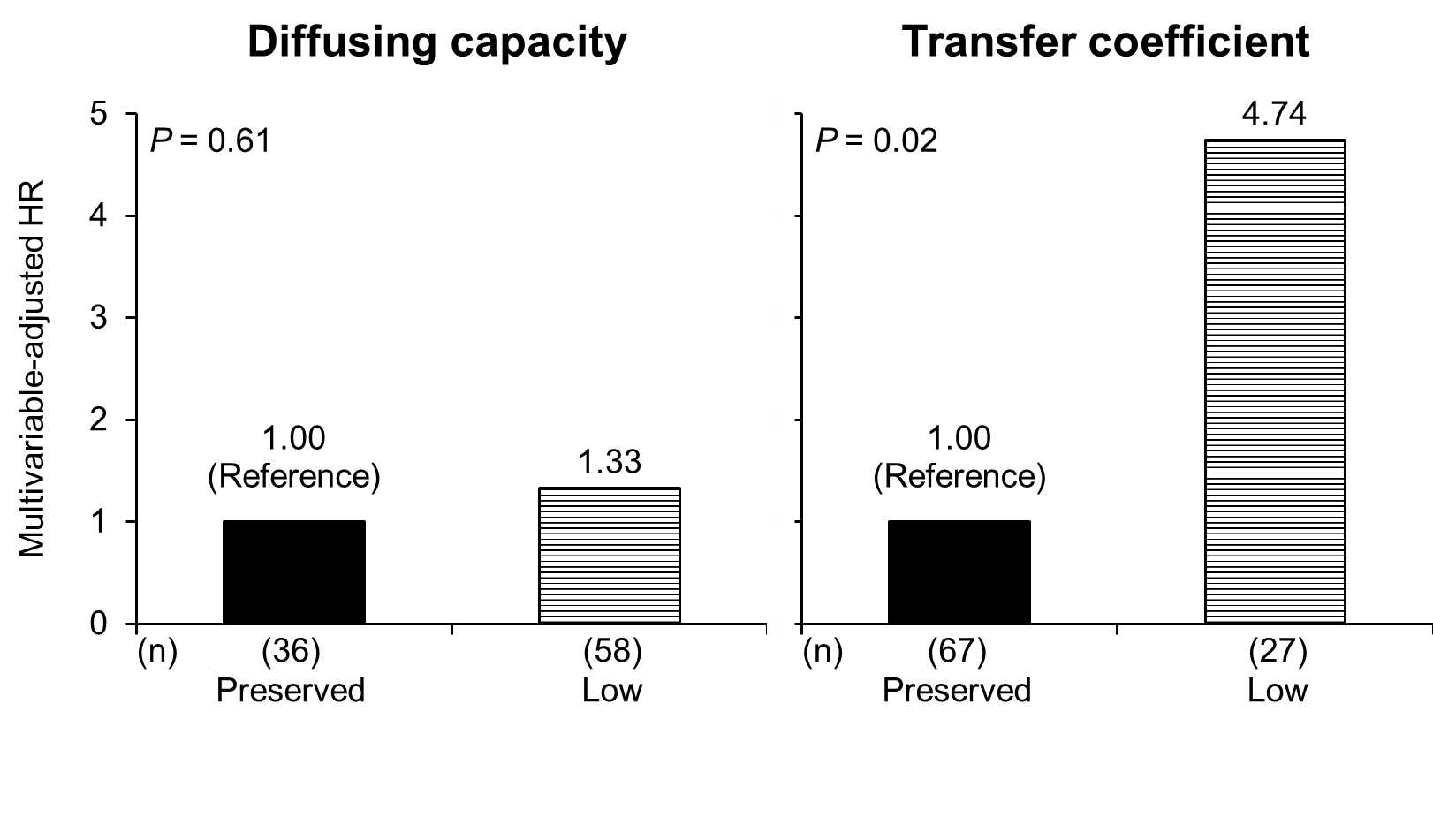


GLI, Global Lung Function Initiative; HR, hazard ratio.

With regard to diffusing capacity, the preserved and low groups consisted of subjects with D_LCO_ % pred ≥ 80% and < 80%, respectively. Similarly, the preserved-K_CO_ and low-K_CO_ group indicated subjects with K_CO_ % pred < 80% and ≥ 80%, respectively.

**e-Figure 6.** The multivariable-adjusted hazard ratios for exacerbation according to the tertile of GLI-based diffusion capacity or transfer coefficient of the lung.


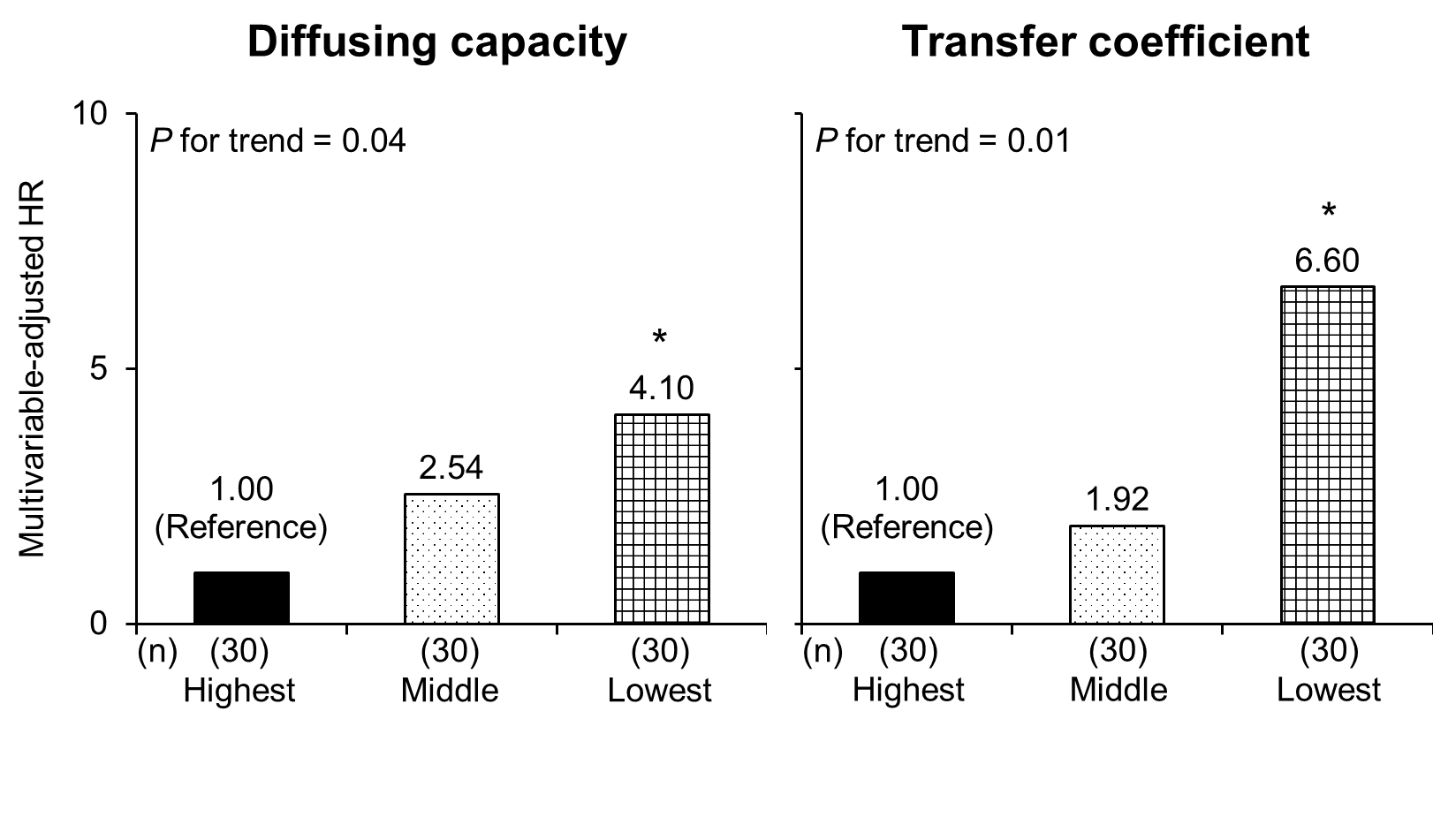


GLI, Global Lung Function Initiative; HR, hazard ratio.

^*^*P* < 0.05 versus the reference group.

With regard to diffusing capacity, the cut-off values for D_LCO_ % pred are indicated as follows: highest, ≥ 81.5%; middle, 62.8–81.4%; and lowest, ≤ 62.7%. Similarly, the cut-offs for K_CO_ % pred were ≥ 102.2% for the highest, 84.0–102.1% for the middle, and ≤ 83.9% for the lowest tertile group.
